# The downstream risk of multiple revisions following a prior TKA revision due to infection is higher compared to an aseptic TKA revision: A nationwide register study

**DOI:** 10.1002/jeo2.70246

**Published:** 2025-04-18

**Authors:** Julius Tetens Hald, Anders Brændsgaard El‐Galaly, Michael Mørk Petersen, Martin Lindberg‐Larsen, Robin Christensen, Anders Odgaard

**Affiliations:** ^1^ Department of Orthopaedic Surgery Rigshospitalet København Ø Denmark; ^2^ Faculty of Health Sciences University of Copenhagen Copenhagen Denmark; ^3^ Department of Orthopaedic Surgery and Traumatology Odense University Hospital Odense Denmark; ^4^ Department of Clinical Research, Orthopaedic Research Unit University of Southern Denmark Sønderborg Denmark; ^5^ Section for Biostatistics and Evidence‐Based Research, The Parker Institute Bispebjerg and Frederiksberg Hospital Copenhagen Denmark; ^6^ Department of Clinical Research, Research Unit of Rheumatology, Odense University Hospital University of Southern Denmark Odense Denmark; ^7^ Department of Clinical Research, Cochrane Denmark & Centre for Evidence‐Based Medicine Odense (CEBMO) University of Southern Denmark Sønderborg Denmark

**Keywords:** infection, knee arthroplasty, multiple revisions, revisions

## Abstract

**Purpose:**

To estimate the downstream effect of septic first revision total knee arthroplasty (rTKA) on the risk of second and third revision compared with aseptic first rTKA.

**Methods:**

A register study of The Danish National Patient Registry and The Danish Knee Arthroplasty Register. rTKAs performed in Denmark from 1998 to 2021 were identified. We included only major revisions defined as the exchange of the tibial and/or femoral components and isolated liner exchanges or patella revisions were not included. Two‐stage revisions were counted as one revision. The exposure group was defined as revisions due to any septic indication (Group 1) while the control group was revisions due to all aseptic indications (Group 2). Cox regression analysis was used to compare the groups and estimate the hazard ratio with 95% confidence interval (95% CI) for revision.

**Results:**

We identified 1016 septic first revisions in Group 1 and 3902 aseptic first revisions in Group 2. The adjusted cox regression analysis demonstrated that Group 1 had a greater risk of a second revision compared to Group 2 (HR 1.4 [95% CI 1.2–1.7]). Additionally, Group 1 had a greater risk of a third revision compared to Group 2 (adjusted HR 1.6 [95% CI 1.1–2.3]).

**Conclusions:**

This is the first study to investigate the downstream risk of revision after a first revision TKA. The adjusted HR of the risk of a third revision was 1.6 higher for septic first rTKAs compared to aseptic first rTKAs, not counting insertions of spacers. This prolonged consequence of PJI is important information for both surgeons and patients.

**Level of Evidence:**

Level III, retrospective comparative study.

**Abbreviations**
CCI: Charlson Comorbidity Index
CCK: condylar constrained knee arthroplasty
DAIR: debridement antibiotics and implant retention
DCRS: Danish Civil Registration System
DKAR: Danish Knee Arthroplasty Register
DNPR: Danish National Patient Register
HR: hazard rate
rTKA: revision total knee arthroplasty
SD: standard deviation
StdD: standardised difference
STROBE: Strengthening the reporting of observational studies in epidemiology
TKA: total knee arthroplasty

## INTRODUCTION

Revision total knee arthroplasty (rTKA) may become necessary following primary total knee arthroplasty (TKA). Recent studies have found that the implant survival of first revisions in Denmark is 75% [3, 9]. The indications for rTKA include infection, aseptic loosening, and instability, among others [15]. Each of these causes is associated with different outcomes but infection has previously been linked to the poorest implant survival rates and identified as a risk factor for re‐revision [10, 13]. This association is primarily attributed to the high recurrence rate of infection, as well as extensive bone and soft tissue loss [8]. Research on patients undergoing multiple knee arthroplasty revisions is limited, but infection is believed to be the predominant cause of multiple revisions [8, 11]. The incidence of multiple revisions, however, is not negligible and seems to lead to increasingly worse implant survival rates [6, 9]. Many studies have shown that the risk of a second revision is high following a first revision TKA due to infection, but no study has investigated the risk of multiple subsequent revisions. Addressing key questions on this topic would qualify the expectations of both surgeons and patients when managing multiple revisions of knee arthroplasties. This knowledge is especially important in specialised revision knee arthroplasty centres which focus on complex revision surgery.

The primary objective was to estimate the comparative risk of the second revision in septic first rTKAs relative to aseptic first rTKAs, observed over a 12‐year period. The secondary objective was to estimate the comparative risk of the third revision in septic first rTKAs, relative to aseptic first rTKAs, observed over a 12‐year period. The hypothesis of this study was: *“The downstream risk of rTKA is determined by the presence of infection at the first revision”*.

## METHODS

### Design and data sources

This is a register study of rTKAs in Denmark from 1 January 1998 to 31 December 2021. Three national databases were used: the Danish National Patient Register (DNPR), The Danish Civil Registration System (DCRS), and the Danish Knee Arthroplasty Register (DKR). The DNPR routinely collects detailed information regarding all Danish residents in the Danish healthcare system such as admissions, surgeries, and outpatient visits since 1977 [17]. All data is linked at a patient level through the Danish Civil Registration number, which is a 10‐digit identifier uniquely assigned to all Danish residents. The DKR is a clinical database which was created in 1997 and since 2006, registration have been mandatory for all Danish hospitals ensuring a completeness above 90% [14]. The surgeon reports detailed surgical information directly after the procedure, such as the type of revision performed, the components used, and the indications for the procedure. Data regarding death and emigration was collected through the DCRS [16]. The study was reported in accordance with the STROBE‐statement [20].

### Study cohort and definitions

The study aimed to include all revisions from rTKAs performed in Denmark from 1 January 1998 to 31 December 2021. To do this, the registers were queried for all primary knee arthroplasty procedures performed from 1 January 1998 to 31 December 2021 in Denmark, based on registrations in both DNPR and DKR [9]. From the primary population, rTKAs were identified. Data on death and emigration were collected. Bilateral arthroplasties were included, so that one patient could contribute with two knees. Thus, the sequence of revisions was secured. A revision was defined as the exchange, addition, or removal of the tibial and/or femoral component. Two‐stage revisions were counted as one revision, with the date of the re‐implantation of the TKA as the date for revision. As such, isolated liner exchanges, including debridement‐, antibiotics, and implant retention (DAIR) procedures, and isolated patella exchanges were excluded from the revision definition. Knee arthrodesis and amputation were also not included. All procedures were grouped based on the indication for rTKA as registered by the surgeon in either DKR or DNPR. In cases of multiple indications given for a procedure, the registration of any infection overruled other indications given [7].

### Study groups (exposures)

The included first rTKA were divided into two groups based on the indication for revision. Group 1 was defined as first revisions registered with the indication confirmed or suspected infection (septic first rTKAs). Group 2—the reference group—was defined as rTKAs registered with a non‐infectious indication (aseptic first rTKAs). These indications included: aseptic loosening, instability, other, pain without loosening, pain, secondary patella and wear. This allocation was kept throughout subsequent revisions, regardless of their indication.

### Outcomes and endpoints

The primary endpoint was the comparative risk of the second revision between Group 1 and Group 2, expressed as hazard ratio (HR) with 95% confidence interval (CI). The secondary endpoint was the comparative risk of the third revision between Group 1 and Group 2, expressed as HR with 95% CI. Follow‐up was limited to 12 years after the first revision due to the increasingly smaller sample size in both groups. Pre‐exposure, baseline variables collected enabled covariate adjustments for biological sex, age, and Charlson Comorbidity Index (CCI) [5].

### Data analyses

Variables that did not appear to follow a normal distribution are presented as medians with interquartile range (IQR). Otherwise, variables are presented as means with standard deviations (SD). Standardised Differences (StdD) were used to quantify differences (with 95% CIs) at baseline for distributions of sex, age, CCI, implant types, and indications for subsequent revisions [4]. Cox regression analyses were performed to compare the groups and estimate HRs with 95% CI. Crude and adjusted HRs were presented with their 95% CI. Schoenfeld plots were used to confirm the proportional hazards assumptions. RStudio version 4.3.1 was used [19].

### Sensitivity analysis

The risk of infections is often driven by systematic risk factors of the individual patient, such as smoking, obesity, diabetes or prior infection in the opposite knee. Therefore, the decision to include bilateral observations might bias the results. To counter this bias, we repeated the analyses including only the first rTKAs of each patient. Secondly, as this study covers nearly three decades of knee arthroplasty, we repeated the analysis using only data from 2010 and onwards to depict whether modern treatment might have affected the results.

## RESULTS

### Baseline characteristics

**Figure 1:**
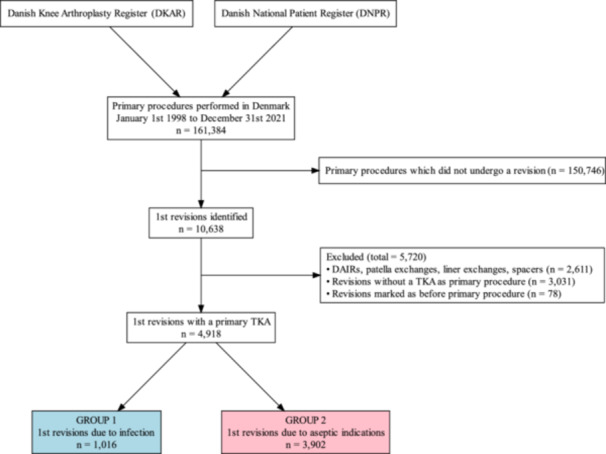
Flowchart of data‐collection. Group 1 marked with blue colour, Group 2 marked with red colour. DAIR, debridement, antibiotics, and implant retention; TKA, total knee arthroplasty.

We identified 161,384 primary TKAs in Denmark from 1 January 1998 to 31 December 2021 (Figure 1). From this cohort, 4918 first TKA revisions with a valid indication were identified. 1016 were due to septic indications (Group 1) and 3902 first revisions were due to aseptic indications (Group 2). There were fewer women (StdD 0.44 [95% CI 0.37–0.51]) and patients were older in group 1 (StdD 0.26 [95% CI 0.19–0.33 years] (Table 1). There was a higher proportion of more comorbid patients in Group 1 as illustrated by the difference in the distribution of CCI scores (StdD 0.25 [95% CI 0.18–0.32]). Finally, there was a higher proportion of more constrained implants in Group 1 as illustrated by the difference in the distribution of implant types (StdD 0.33 [95% CI 0.26–0.40]).

**Table 1:** Baseline characteristics (at time of the first revision) grouped by the indication of the first rTKA.

	Total, *n* = 4918 (100%)	Group 1, *n* = 1016 (21%)	Group 2, *n* = 3902 (79%)	Difference [95% CI]^a^
Women	2982/4918 (61)	443/1016 (44)	2539/3902 (65)	0.44 [0.37–0.51]
Age, years	67 (59–74)	70 (62–76)	67 (59–74)	0.26 [0.19–0.33]
CCI				0.25 [0.18–0.32]
0	2426/4918 (49)	455/1016 (45)	1971/3902 (51)	
1	1023/4918 (21)	173/1016 (17)	850/3902 (22)	
2	686/4918 (14)	159/1016 (16)	527/3902 (14)	
>=3	783/4918 (16)	229/1016 (23)	554/3902 (14)	
Type of implant				0.33 [0.26–0.40]
Unconstrained^b^	1501/4918 (31)	200/1016 (20)	1301/3902 (33)	
CCK	2840/4918 (58)	666/1016 (66)	2174/3902 (56)	
Hinge	409/4918 (8)	94/1016 (9)	315/3902 (8)	
Unknown	168/4918 (3)	56/1016 (5)	112/3902 (3)	

*Note*: Group 1 = first rTKAs due to infection. Group 2 = first rTKAs due to aseptic causes. Categorical values are given as *n* with %. Numerical values are given as medians with interquartile range.

Abbreviations: CCI, Charlson Comorbidity Index; CCK, condylar constrained knee; CI, confidence interval; rTKA, revision total knee arthroplasty.

^a^ Standardised differences (StdD) for categorical and numerical variables.

^b^ Unconstrained implants include cruciate‐retaining and posterior stabilised implants.

### Indications for second and third revisions

We identified 712 second revisions and 166 third revisions from the two groups (Table 2). There was a significant difference in the indications for the second revisions between the groups. In Group 1, 54% (91/167) of the second revisions were registered due to infection, while the corresponding number for Group 2 was 13% (73/545) (StdD 0.96 [95% CI 0.78–1.14]). This difference was still present in the third revision: 42% (19/45) of the third revisions in Group 1 were due to infection, while the corresponding number for Group 2 was 19% (23/121) (StdD 0.52 [95% CI 0.17–0.87].

**Table 2:** Incidence of revisions.

	Total, *n* = 4918 (100%)	Group 1, *n* = 1016 (21%)	Group 2, *n* = 3902 (79%)	Difference [95% CI]^a^
Second revisions	712/4918 (14)	167/1016 (16)	545/3902 (14)	0.96 [0.78–1.14]
Infection	164/712 (23)	91/167 (54)	73/545 (13)	
Aseptic	548/712 (77)	76/167 (46)	472/545 (87)	
Third revisions	166/4918 (3.4)	45/1016 (4.4)	121/3902 (3.1)	0.52 [0.17–0.87]
Infection	42/166 (25)	19/45 (42)	23/121 (19)	
Aseptic	124/166 (75)	26/45 (58)	98/121 (81)	
Follow up period, years^b^	6.4 (3–10)	6.1 (3–10)	6.5 (3–10)	0.05 [−0.02–0.12]

*Note*: Group 1 = first rTKAs due to infection. Group 2 = first rTKAs due to aseptic causes. Categorical values are given as *n* with %. Numerical values are given as medians with interquartile range.

Abbreviations: CI, confidence interval; rTKA, revision total knee arthroplasty.

^a^ Standardised differences (StdD) for categorical and numerical variables.

^b^ Follow‐up period was defined as the time from first revision to death, emigration or end of observation period.

### Risks of the second and third revisions

The Schoenfeld plots confirmed that the proportional hazards assumption was met. Cox regression demonstrated that Group 1 had a higher crude risk of a second revision compared to Group 2 (HR 1.2 [95% CI 1.0–1.5]) (Table 3). This remained statistically significant when adjusting for sex, age, CCI and implant type (HR 1.4 [1.2–1.7]). Increasing age in years decreased the risk of a second revision by an HR of 0.97 [0.97–0.98]. Knees with a CCK implant at the time of the first revision had a lower risk of a second revision (HR 0.8 [95% CI 0.7–0.9]) compared to knees with an unconstrained implant. Knees with a hinged implant had a lower risk of a second revision (HR 0.6 [95% CI 0.4–0.8]).

**Table 3:** Cox regression of the risk on the second revision.

Values are given as hazard ratios, relative to Group 2 [95% CI]					
Group 1^a^	1.2 [1.0–1.5]	1.2 [1.0–1.4]	1.3 [1.1–1.6]	1.3 [1.1–1.6]	1.4 [1.2–1.7]
Men^b^		1.1 [0.96–1.3]	1.1 [0.9–1.2]	1.1 [0.9–1.2]	1.0 [0.9–1.2]
Age, years^c^			0.97 [0.96–0.98]	0.97 [0.97–0.98]	0.97 [0.97–0.98]
CCI – 1^d^				1.0 [0.8–1.2]	1.0 [0.9–1.3]
CCI – 2				0.9 [0.7–1.2]	0.9 [0.7–1.2]
CCI – 3				0.9 [0.7–1.2]	1.0 [0.8–1.2]
CCK^e^					0.8 [0.7–0.9]
Hinge					0.6 [0.4–0.8]

*Note*: Increasing control for covariates. Values given in the left of the column represent unadjusted hazard ratios. The columns to the right represent the hazard ratios with stepwise adjustment of covariates. Group 1 = first rTKAs due to infection. Group 2 = first rTKAs due to aseptic causes.

Abbreviations: CCI, Charlson Comorbidity Index; CCK, condylar constrained knee; CI, confidence interval; rTKA, revision total knee arthroplasty.

^a^ Group 2 as reference.

^b^ Sex as a dichotomous variable. Women as reference.

^c^ Age as a continuous variable.

^d^ Charlson Comorbidity Index as a categorical variable. 0 as reference.

^e^ Implant type as a categorical variable. Unconstrained as reference.

Infection at the time of the first revision increased the risk of any third revision by a crude HR of 1.5 [95% CI 1.1–2.1] (Table 4). Again, this remained statistically significant with adjustment of sex, age, CCI and implant type by an HR of 1.6 [95% CI 1.1–2.3]. Men were found to have a higher HR of a third revision by 1.5 [95% CI 1.1–2.1]. Increasing age in years decreased the risk of a third revision by an HR of 0.97 [0.96–0.98]. Knees with a CCK implant at the time of the first revision had a lower risk of a third revision by an HR of 0.7 [95% CI 0.5–0.99] compared with unconstrained implants.

**Table 4:** Cox regression of the risk of the third revision.

Values are given as hazard ratios, relative to group 2 [95% CI]					
Group 1^a^	1.5 [1.1–2.1]	1.3 [0.9–1.9]	1.5 [1.1–2.1]	1.5 [1.1–2.2]	1.6 [1.1–2.3]
Men^b^		1.6 [1.2–2.2]	1.5 [1.1–2.1]	1.5 [1.1–2.1]	1.5 [1.1–2.1]
Age, years^c^			0.97 [0.95–0.98]	0.97 [0.96–0.98]	0.97 [0.96–0.98]
CCI – 1^d^				1.0 [0.7–1.4]	1.0 [0.7–1.5]
CCI – 2				0.6 [0.4–1.1]	0.7 [0.4–1.2]
CCI – 3				0.7 [0.4–1.2]	0.7 [0.4–1.2]
CCK^e^					0.7 [0.5–0.99]
Hinge					0.6 [0.3–1.3]

*Note*: Increasing control for covariates. Values given in the left of the column represent unadjusted hazard ratios. The columns to the right represent the hazard ratios with stepwise adjustment of covariates. Group 1 = first rTKAs due to infection. Group 2 = first rTKAs due to aseptic causes.

Abbreviations: CCI, Charlson Comorbidity Index; CCK, condylar constrained knee; CI, confidence interval; rTKA, revision total knee arthroplasty.

^a^ Group 2 as reference.

^b^ Sex as a dichotomous variable. Women as reference.

^c^ Age as a continuous variable.

^d^ Charlson Comorbidity Index as a categorical variable. 0 as reference.

^e^ Implant type as a categorical variable. Unconstrained as reference.

### Sensitivity analysis

When repeating the analyses, excluding the second arthroplasty performed in bilateral rTKAs, our results did not differ from our main analysis. The adjusted HR for a second revision was 1.4 [95% CI 1.2–1.7] for group 1 and the adjusted HR for a third revision was 1.6 [95% CI 1.1–2.3] for Group 1. When repeating the analyses, only including rTKAs performed from 1 January 2010, and onwards, our results did not differ from our main analysis which indicated robustness of the main analyses. The adjusted HR for a second revision was 1.5 [95% CI 1.2–1.8] for Group 1 and the adjusted HR for a third revision was 1.8 [95% CI 1.1–2.9] for Group 1.

## DISCUSSION

The most important finding of this study was that the adjusted HR of the risk of a third revision was 1.6 higher for septic first rTKAs compared to aseptic first rTKAs, not counting insertions of spacers. The adjusted HR of the risk of a second revision was 1.4 higher for septic first rTKAs compared to aseptic first rTKAs. Our results confirmed our hypothesis that the indication of first rTKA was associated with the downstream risk of multiple subsequent revisions. To our knowledge, this is the first study to investigate the downstream risk of a third revision following a septic first rTKA.

In this study, we provide the first evidence on the risk of downstream revisions. While some studies have investigated the risk of failure after a revision TKA, none have reported the risk of failure of re‐revisions stratified by the indications for the procedures. The Danish health registers serve as optimal tools for this investigation, as they provide coverage of all knee arthroplasty patients in Denmark and complete follow‐up [16, 17, 18]. This enables reliable identification of all relevant patients over a long observation period without loss to follow‐up. Additionally, it allows us to identify third rTKAs, which is challenging in health insurance‐based registries. Few studies have investigated multiple revisions of knee arthroplasties, but in general, the survivability of the implant decreases dramatically for each consecutive revision [6, 9]. This decrease in implant survivability is even greater in cases of rTKA due to infection [12, 13]. Two previous studies have found corresponding risks for a second revision following a first rTKA due to infection compared to aseptic first rTKAs with an odds ratio of 1.9 and a relative risk of 2.7, respectively [1, 8]. A systematic review has investigated the risk factors for re‐revision following revision TKA. The study identified 26 risk factors for re‐revision [10]. The review identified four studies which all found a statistically significant risk factor for re‐revision when the index revisions were performed due to infection, compared to index revisions performed due to aseptic causes [10]. However, the studies are limited by a small sample size, short follow‐up, and lacking multiple centres. Still, more evidence is mounting for the argument that multiple revision knee arthroplasties revised for infection fail more frequently than multiple revision knee arthroplasties revised for aseptic causes [8, 12]. Reinfection is argued by some studies to be the most common cause of failure which often occurs within the initial years following the revision [8, 12] and we also found that infection continued to be the main indication for the second and third revision, if the first revision was due to infection. It has been shown that the largest difference in survivability between septic and aseptic rTKAs occurs in the first two years [13]. In this study, we chose to exclude DAIR‐procedures and count two‐stage revisions as one. It is possible that the difference in risk between septic and aseptic first rTKAs would be greater in a study focusing on acute infections and DAIRs.

### Limitations

There are some limitations in this study. The main limitation consists of the data used for classifying infections; these were surgeon‐reported and not based on microbiological data, which might give a more precise estimate. A sensitivity study of the DKR has shown that surgeons in Denmark tend to underreport infections by up to 36%, meaning that our data potentially underestimates the proportion of revisions due to infection [2]. Yet, culture‐negative infections are well‐known in arthroplasty surgery. Thus, relying on the surgical indication for revision approximates the real‐world clinical setting. Additionally, there has been some evidence that the incidence of septic rTKAs has been increasing in the late period of the observation period [12]. However, our sensitivity analysis demonstrated that this was not a problem as we found no differences in our results when limiting the analysis to rTKAs performed in 2010 and onwards. The inclusion of bilateral knees did not confound our results as our sensitivity analysis confirmed the results of our main analysis.

## CONCLUSION

In conclusion, this study found that infection at the time of the first rTKA increased the risk of a second revision by an HR of 1.4 and of a third revision by an HR of 1.6. The associated downstream risk of a third revision, for patients revised the first time due to infection, was significantly higher compared to patients revised the first time due to aseptic indications. This study provides new evidence to revision knee arthroplasty surgeons; *first rTKAs due to infection have greater risks of multiple downstream revisions than other first rTKAs*.

## AUTHOR CONTRIBUTIONS

**Julius Tetens Hald**: Conceptualisation; methodology; validation; formal analysis; investigation; data curation; writing—original draft; writing—review and editing; visualisation; project administration. **Anders Brændsgaard El‐Galaly**: Conceptualisation; methodology; validation; writing—review and editing; supervision. **Michael Mørk Petersen**: Conceptualisation; methodology; writing—review and editing; supervision. **Martin Lindberg‐Larsen**: Conceptualisation; methodology; writing–review and editing; resources; supervision. **Robin Christensen**: Conceptualisation; methodology; validation; writing—review and editing; supervision. **Anders Odgaard**: Conceptualisation; methodology; writing—review and editing; resources; supervision; funding acquisition.

## CONFLICT OF INTEREST STATEMENT

The authors declare no conflicts of interest.

## ETHICS STATEMENT

Prior to data collection, the study was approved by the Danish Data Protection Agency through the Capitol Region of Denmark (case number: P‐2022‐711). Register studies in Denmark do not require approval from the regional ethical committee.

## DECLARATION

During the preparation of this work the author(s) used chatgpt.org in order to check spelling and grammar. After using this tool/service, the author(s) reviewed and edited the content as needed and take(s) full responsibility for the content of the publication.

## Data Availability

The data used in this study is classified as personally identifiable data, and thus, it is illegal to forward data in any way without prior approval from the relevant Danish data agencies.

## References

[1] Aggarwal VK , Goyal N , Deirmengian G , Rangavajulla A , Parvizi J , Austin MS . Revision total knee arthroplasty in the young patient: is there trouble on the horizon? J Bone Joint Surg Am. 2014;96:536–542. 24695919 10.2106/JBJS.M.00131

[2] Anneberg M , Kristiansen EB , Troelsen A , Gundtoft P , Sørensen HT , Pedersen AB . Enhancing the data capture of periprosthetic joint infections in the Danish Knee Arthroplasty Registry: validity assessment and incidence estimation. Acta Orthop. 2024;95:166–173. 38595072 10.2340/17453674.2024.40358 PMC11004670

[3] Arndt KB , Schrøder HM , Troelsen A , Lindberg‐Larsen M . Prosthesis survival after revision knee arthroplasty for “pain without loosening” versus “aseptic loosening”: a Danish nationwide study. Acta Orthop. 2022;93:103–110. 34906032 10.1080/17453674.2021.1999069 PMC8815427

[4] Austin PC . Balance diagnostics for comparing the distribution of baseline covariates between treatment groups in propensity‐score matched samples. Stat Med. 2009;28:3083–3107. 19757444 10.1002/sim.3697 PMC3472075

[5] Charlson ME , Pompei P , Ales KL , MacKenzie CR . A new method of classifying prognostic comorbidity in longitudinal studies: development and validation. J Chronic Dis. 1987;40:373–383. 3558716 10.1016/0021-9681(87)90171-8

[6] Deere K , Whitehouse MR , Kunutsor SK , Sayers A , Price AJ , Mason J , et al. How long do revised and multiply revised knee replacements last? A retrospective observational study of the National Joint Registry. Lancet Rheumatology. 2021;3:e438–e446. 35043097 10.1016/S2665-9913(21)00079-5 PMC7612217

[7] El‐Galaly A , Grazal C , Kappel A , Nielsen PT , Jensen SL , Forsberg JA . Can machine‐learning algorithms predict early revision TKA in the Danish Knee Arthroplasty Registry? Clin Orthop Relat Res. 2020;478:2088–2101. 32667760 10.1097/CORR.0000000000001343 PMC7431253

[8] Geary MB , Macknet DM , Ransone MP , Odum SD , Springer BD . Why do revision total knee arthroplasties fail? A single‐center review of 1632 revision total knees comparing historic and modern cohorts. J Arthroplasty. 2020;35:2938–2943. 32561262 10.1016/j.arth.2020.05.050

[9] Hald JT , El‐Galaly AB , Petersen MM , Lindberg‐Larsen M , Christensen R , Odgaard A . Incidence and survival of multiply revised knee arthroplasties in Denmark 1998‐2021: a nationwide register‐based study. Acta Orthop. 2024;95:454–459. 39167020 10.2340/17453674.2024.41257 PMC11337950

[10] Hald JT , Knudsen UK , Petersen MM , Lindberg‐Larsen M , El‐Galaly AB , Odgaard A . Risk factors associated with re‐revision following revision total knee arthroplasty: a systematic review. Bone Joint Open. 2024;5:644–651. 39106978 10.1302/2633-1462.58.BJO-2024-0073.R1 PMC11303039

[11] Klasan A , Magill P , Frampton C , Zhu M , Young SW . Factors predicting repeat revision and outcome after aseptic revision total knee arthroplasty: results from the New Zealand Joint Registry. Knee Surg Sports Traumatol Arthrosc. 2021;29:579–585. 32279110 10.1007/s00167-020-05985-8

[12] Leta TH , Lygre SHL , Schrama JC , Hallan G , Gjertsen JE , Dale H , et al. Outcome of revision surgery for infection after total knee arthroplasty: results of 3 surgical strategies. JBJS Rev. 2019;7:e4. 10.2106/JBJS.RVW.18.00084 31188156

[13] Mortazavi SMJ , Molligan J , Austin MS , Purtill JJ , Hozack WJ , Parvizi J . Failure following revision total knee arthroplasty: infection is the major cause. Int Orthop. 2011;35:1157–1164. 20959981 10.1007/s00264-010-1134-1 PMC3167421

[14] Pedersen A , Mehnert F , Odgaard A , Schrøder HM . Existing data sources for clinical epidemiology: the Danish Knee Arthroplasty Register. Clin Epidemiol. 2012;4:125–135. 22701092 10.2147/CLEP.S30050 PMC3372970

[15] Postler A , Lützner C , Beyer F , Tille E , Lützner J . Analysis of total knee arthroplasty revision causes. BMC Musculoskelet Disord. 2018;19:55. 29444666 10.1186/s12891-018-1977-y PMC5813428

[16] Schmidt M , Pedersen L , Sørensen HT . The Danish Civil Registration System as a tool in epidemiology. Eur J Epidemiol. 2014;29:541–549. 24965263 10.1007/s10654-014-9930-3

[17] Schmidt M , Schmidt SAJ , Sandegaard JL , Ehrenstein V , Pedersen L , Sørensen HT . The Danish National Patient Registry: a review of content, data quality, and research potential. Clin Epidemiol. 2015:449–490. 26604824 10.2147/CLEP.S91125 PMC4655913

[18] Schmidt M , Schmidt SAJ , Adelborg K , Sundbøll J , Laugesen K , Ehrenstein V , et al. The Danish health care system and epidemiological research: from health care contacts to database records. Clin Epidemiol. 2019;11:563–591. 31372058 10.2147/CLEP.S179083 PMC6634267

[19] *R: A language and environment for statistical computing* [computer program]. 2021.

[20] Vandenbroucke JP , Elm E , Altman DG , Gøtzsche PC , Mulrow CD , Pocock SJ , et al. Strengthening the Reporting of Observational Studies in Epidemiology (STROBE): explanation and elaboration. Ann Intern Med. 2007;147:W163–W194. 17938389 10.7326/0003-4819-147-8-200710160-00010-w1

